# Roots of sustainability

**DOI:** 10.1007/s00709-022-01798-3

**Published:** 2022-07-30

**Authors:** Peter Nick

**Affiliations:** Botanical Institute, Karlsruhe Institute of Technology, Karlsruhe, Germany

Latest since the days of the Titanic or Sigmund Freud (1915), we know that the things underneath the surface are often more relevant than that what is visible. This holds true for agriculture as well. The soil that is currently used or has been formerly used for agriculture is able to host around half of the entire carbon dioxide that has been generated by human activity so far—on the condition that we treat it well (Lal 2004). Likewise, it is mainly the root that is crucial for stress resilience of plants. For instance, root architecture can adapt in a flexible and sophisticated manner to forage local sources of water or nutrients, such as phosphorous (Lopez-Bucio et al. 2002). The root is not alone, but intimately intertwined with specific microbial communities that contribute to the well-being of the plant by specific metabolites. Some of these metabolites act as signals supporting adaptation to environmental stresses (for a recent review see Hong et al. 2022). For the specific microbial ecosystem surrounding a root, the term *rhizosphere* has been coined (Hiltner 1904). Two contributions to the current issue take the reader downsoil to collect some insights into the secrets of the underworld that is as important for our survival on this planet as it is unknown.

Phosphorus (P) is central to biology—there would neither be ATP, nor DNA without phosphate. Fertilising inorganic phosphate has been a main driver for Green Revolution, therefore. However, the phosphorous mines will become depleted within a few decades from now, rising progressive political concern on the sustainability of current agriculture (Haarr 2005). In principle, P is abundant, but since it is bound in immobile complexes, it is only poorly available for plants. To scavenge the low concentrations of inorganic phosphate, they have to rely on potent phosphate transporters, which is often the limiting factor in many cereals, such as wheat, progressively posing serious risks upon global food security. In their contribution to the current issue, Li et al. (2022) demonstrate, how a regulator from rice, PHOSPHATE RESPONSE 2, can boost phosphorous accumulation in wheat, especially under conditions of limited P supply. This factor belongs to the widespread family of MYB transcription factors and is activated upon phosphate starvation, deploying a plethora of downstream genes, including phosphate transporters. The authors inserted this master switch under control of its own promoter, such that it would become active exclusively when needed, under conditions of phosphate limitation. While the regulator was mainly expressed in leaves, it had a strong effect on root development, especially under phosphate starvation. The root system was not only longer, but displayed a higher degree of ramification, which improves the interaction between root and soil. The richer root architecture was accompanied by a significant increase in grain yield. This work demonstrates, for the important staple crop bread wheat, that breeding for improved phosphate acquisition represents a sustainable alternative for mineral fertiliser.

Changes in root architecture cannot only be induced by abiotic factors, such as phosphate starvation, but also in response microbial signals. This is addressed in the contribution by García-Cárdenas et al. (2022). These authors had identified the phytohormone auxin as important target for microbial manipulation. For instance, they had shown in previous work that a bacterium isolated from the maize rhizosphere, *Bacillus methylotrophicus* strain M4-96, emits volatile signals that induce auxin biosynthesis in a thale cress recipient, such that the recipient plant grew faster (Pérez-Flores et al. 2017). Also root architecture has been found to be modulated via auxin status, for instance by *Azospirillum brasilense* (Méndez-Gómez et al. 2021). In the current work, these authors addressed the cellular mechanism behind such architectural responses of root systems, identifying a Gram-positive bacterium as signal donor using *Arabidopsis thaliana* as recipient model. Starting with a maize rhizosphere community from an alkaline soil as source, they conducted a bioactivity-guided screen for growth promotion and identified *Micrococcus luteus* strain LS570 as signal donor. This donor promotes root branching and, thus, the absorptive potential of the root system. Using auxin-responsive promoter reporter lines, they could show that the presence of this bacterium activated auxin signalling, and a detailed anatomical investigation revealed that apical dominance of the primary root became inhibited linked with reduced mitotic activity in the meristem and reduced cell expansion in the distal elongation zone. The observed root branching seems to be a secondary consequence, though, because a drop of apical dominance will activate the pericycle to form more lateral roots. By the use of mutants impaired in individual auxin response factors, the authors could even identify ARF7 and ARF19 as responsible factors. Thus, this bacterium modulates auxin signalling and, in consequence, the morphogenetic responses under control of auxin signalling. A richer and more ramified root system not only improves the uptake of nutrients, but is also a hallmark for resilience, for instance to drought stress. Thus, this work paves the way for engineering the rhizosphere in a way that the performance of crop plants under stress is improved.

Both contributions are not only of relevance for a more sustainable agriculture, but also demonstrate that understanding the cellular mechanisms of a phenomenon can help to develop better solutions for application. Cell biology is often (mis)understood as a paradigm for fundamental science that often appears far from practical relevance. This viewpoint is definitely wrong. Although it may be possible to engineer solutions by mere optimisation without a clear understanding of the underlying mechanisms, this approach will run into problems in the long term, because troubleshooting will become very cumbersome, as soon as a certain level of complexity is reached. Applications that are based on a sound understanding of the causal relationships responsible for the phenomenon will be easier to be adjusted to new challenges. In other words: hypothesis-driven application is more sustainable.
